# Understanding the maternal sepsis patient journey in Malawi: “I called for help, but they showed no interest in helping me”

**DOI:** 10.1186/s12913-025-13459-1

**Published:** 2025-09-30

**Authors:** Yamikani Chimwaza, Chikondi Chapuma, Chifundo Ndamala, Emily Lifa, Mercy Machilika, Bernard Dossie, Meliya Kwelepeta, Bertha Maseko, David Lissauer, Alinane Linda Nyondo-Mipando, Maria Lisa Odland

**Affiliations:** 1 Malawi Liverpool Wellcome Programme, P.O. Box 30096, Blantyre, Malawi; 2 Department of Women’s and Children’s Health, Institute of Life Course and Medical Sciences, University of Liverpool, Liverpool, UK; 3 Kamuzu University of Health Sciences, Blantyre, Malawi

**Keywords:** Maternal sepsis, Patient journey mapping, Experience of care, Three Delays model

## Abstract

**Background:**

In Malawi, there is a limited understanding of the personal experiences of women who have survived severe maternal infections that led to sepsis and their perspectives on the care they received. It is essential to consider women’s experiences to improve the quality of care for severe maternal outcomes and provide responsive, patient-centered care. This study aimed to explore the experiences of maternal sepsis survivors in Malawi to improve sepsis care and management.

**Methods:**

A qualitative descriptive design study was conducted from April to May 2023 in Blantyre, Malawi. Pregnant, postpartum, and postabortion women older than 16 years who had sepsis were included in the study via purposive sampling. Face-to-face interviews were conducted at the women’s homes or convenient locations. A thematic analysis was performed using the Three Delays and Respectful Maternity Care frameworks to analyse the interview data.

**Results:**

Women with sepsis faced multiple barriers to accessing care, mainly delays in receiving adequate and appropriate care at health facilities. The key barriers mentioned by participants highlighted the inadequate monitoring, inadequate clinical management of infections, delays in diagnosis and treatment, and delays in providing lifesaving obstetric skills at the health facility. Some women also experienced mistreatment by healthcare providers, such as verbal abuse, neglect, abandonment, lack of dignity, disrespect, denial of care, and inequitable treatment. Additionally, some women continue to experience long-term physical and psychological complications from maternal sepsis.

**Conclusion:**

Understanding patient experiences is essential for improving the quality of maternal sepsis care and management. Patient narratives can inform policy and maternal healthcare, highlighting the need for feedback-based systems. These insights are relevant to other resource-constrained settings facing high maternal infection rates. Comprehensive, patient-centred interventions are crucial for managing maternal infections which lead to sepsis, thus reducing preventable morbidity and mortality.

**Supplementary Information:**

The online version contains supplementary material available at 10.1186/s12913-025-13459-1.

## Background

Every day, almost 800 women die from preventable causes related to pregnancy and childbirth [1]. Nearly 95% of all maternal deaths occurred in low- and lower-middle-income countries (LMICs) in 2020 [1]. Globally, maternal sepsis represents the third most prevalent cause of maternal mortality, following postpartum haemorrhage and preeclampsia [2, 3]. Maternal sepsis is a life-threatening condition that occurs when the body’s response to infection causes injury to tissues and organs during pregnancy, childbirth, postabortion, or the postpartum period [4]. In Malawi, infections during pregnancy are the primary contributors to maternal mortality, responsible for 24.8% of all such deaths [5]. Sepsis poses a significant threat to maternal health in Malawi, with research from decades ago emphasizing its prevalence and the difficulties encountered by healthcare providers in managing it. In 2005, research conducted at Queen Elizabeth Central Hospital (QECH) identified sepsis as the leading cause of maternal deaths (29.4%) [6]. A 2016 evaluation of 36 health facilities revealed critical resource gaps in implementing sepsis management guidelines, exacerbating mortality rates [7, 8]. The Malawi Confidential Enquiry into Maternal Deaths (2008–2012) noted delays in recognising and treating maternal sepsis while recommending structured monitoring, early warning scores, and provider education [9]. Clinical trials are ongoing to enhance sepsis recognition, vital sign monitoring, and antibiotic administration in government facilities, aiming to improve infection prevention nationwide [10, 11]. Despite these efforts, maternal sepsis remains a significant concern, necessitating sustained investment in healthcare infrastructure, provider training, and standardised protocols to reduce maternal deaths.

Traditionally, assessing women’s health and the quality of obstetric care has involved analysing cases to identify areas for improvement [12]. Although this approach provides valuable insights into maternal healthcare standards in various settings [13], it can also instil fear among healthcare providers. They may worry that their involvement in these cases could lead to blame or legal repercussions, diminishing the chances for open and sincere discussions. Women who become critically ill during pregnancy or childbirth but survive are termed “near-miss cases” [14]. Examining these cases allows survivors to share their experiences, providing valuable perspectives on healthcare. This approach facilitates learning and encourages open discussions about patient care from the patient’s point of view, potentially contributing to improvements in maternal healthcare. The focus on quality includes an expanding emphasis on women’s experiences of care and the importance of delivering care that honours the mother’s inherent dignity [15]. A positive experience of care is associated with better physical and mental health outcomes [16]. Women’s experiences with life-threatening conditions such as sepsis and their experiences with their healthcare providers can empower or comfort them or inflict lasting damage and emotional trauma [17].

Maternal sepsis significantly affects pregnant women, resulting in grave social and economic consequences for families and communities. The challenges intensify when a mother (the primary caregiver) endures extended hospitalisation, a lengthy recovery, or even death, disrupting family stability, particularly for children [18]. Consequently, children may face poorer health outcomes and limited educational opportunities, especially older siblings who must take on maternal roles and have an elevated risk of neglect. Economically, families often confront daunting medical expenses and the loss of a mother’s productivity and contributions to the household and community economy, leading to financial instability and entrenching cycles of poverty [19].

Malawi has made notable progress in improving access to healthcare services; however, challenges remain in achieving improved outcomes related to the quality of care [20]. Acknowledging this, the Malawi Ministry of Health established the Quality Management Directorate to spearhead initiatives to elevate healthcare quality. The current policy focuses on strengthening leadership, governance, and accountability, enhancing clinical skills and competencies, improving patient safety, encouraging people-centred care, and developing research and monitoring capabilities [20]. Although advancements have been made, healthcare quality reform often overlooks the patient experience. Incorporating patient insights into quality improvement efforts is crucial to providing clinically effective care and respectfully responding to individuals’ needs. To improve the quality of care for preventing and managing maternal sepsis, it is imperative to understand the patient’s journey, identify any delays or barriers in diagnosing, treating, or recovering from sepsis and provide responsive, patient-centred care. This study explored the personal experiences of maternal sepsis survivors in Malawi to deepen our understanding of their healthcare journeys, including barriers and delays in diagnosis, treatment, and recovery. By adopting a qualitative approach, the study amplified the voices of sepsis survivors, offering insights into their perceptions of care and highlighting areas where healthcare systems can improve.

## Methods

### Study design

A qualitative descriptive design study was conducted in Blantyre, Malawi, from April to May 2023. The study focused on women who were pregnant, postpartum (within six weeks of pregnancy outcome) or post-abortion, who were within eight weeks of hospital discharge. These women were 16 years old or older. They had previously participated in the QECH study titled “Using lactate testing to improve maternal sepsis identification: a multicountry test accuracy study: LACTate in mATernal sEpsis (LACTATE)” from July 2022 to June 2023. The Lactate study was a prospective, multisite, phase III trial to assess the diagnostic accuracy of maternal lactate measurements and maternal vital sign thresholds for sepsis. Additionally, this study aimed to identify women at risk of severe morbidity or mortality in low-resource settings [11].

### Study setting

The Queen Elizabeth Central Hospital in Blantyre is the oldest and largest government-owned tertiary facility for outpatient department attendance, hospital admissions, and specialist services in Malawi’s commercial capital. It provides tertiary services to the entire population of southern Malawi, which is expected to exceed 8 million [21]. The Gogo Chathinkha wing at QECH specialises in maternal and child health services, including antenatal, postnatal, gynaecological, labour and delivery, a high dependency unit, and an operating theatre. Between April 2023 and March 2024, there were a total of 10,774 deliveries and 52 maternal deaths at QECH [22].

### Sampling and sample size

We reviewed case report forms from the Lactate Study Database to identify potential sepsis cases based on admission reasons, severe complications during hospitalisation, and laboratory results. We employed purposive sampling methods to recruit women who met the criteria for maternal sepsis (i.e., infection and organ dysfunction), ensuring the selection of women with a diverse range of maternal infections (such as pneumonia, chorioamnionitis, septic abortion, and urinary tract infection) and various pregnancy outcomes that are representative of the post-abortion, postpartum, and pregnant periods. We aimed to collect experiences from up to 20 sepsis survivors to capture unique patient experiences in maternal sepsis care. Our sample size was sufficient because of the saturation of ideas, information power, theoretical analysis models, and engaging interview techniques, especially considering the lack of previously described experiences of maternal sepsis survivors in low-resource settings.

### Recruitment of study participants

The research team proposed this study to potential participants during follow-up in the Lactate study or by phone (study nurses obtained participant contact information during the Lactate enrollment visit) after exiting the Lactate study. Interested Lactate participants were given time to consider participation in the study. As former study participants, these women were acquainted with the study team and receptive to participating in the in-depth interviews. Identifying and locating these women was made easier by having previously collected participant contact information at hand. Additionally, these women consented to future contact after leaving the lactate study. Women provided consent at their lactate study exit visit or before the interview.

### Data collection

Face-to-face interviews were conducted using an Inpatient Assessment of Healthcare (I-PAHC) questionnaire (Supplementary file 1) to measure patient healthcare experiences, followed by audio-recorded in-depth interviews. The IPAHC is a brief survey tool developed by Webster et al. and validated in Ethiopia to explore experiences of care in low-income settings [23]. Before the data collection phase began, the translated interview tools were tested and piloted on three randomly selected pregnant and postpartum inpatients from the QECH maternity wards to ensure their effectiveness in gathering information. The tools were revised and improved as needed. The pilot interview results were not included in the analysis. The interviews took place at participants’ homes or other convenient locations to ensure privacy. Experienced research nurses (MM, EM, BD, and MM) used a semi-structured interview guide (Supplementary file 2). The interviews and discussions lasted 30 to 60 min on average. Data saturation was reached at the 9th in-depth interview when we noted a repetition of themes, and no new themes, patterns or insights emerged. Real-time coding and monitoring of the transcripts were conducted during data collection to track emerging themes and identify when saturation was achieved. The team conducted four additional interviews to confirm saturation and that the data sufficiently addressed the study objectives before concluding the data collection phase. In the case of one participant who was unconscious for a significant period during hospitalisation, information was gathered from her grandmother, who acted as her guardian. This was done to enhance the credibility of the patient’s findings during the interview.

### Data analysis

All hardcopy data were kept in a locked cabinet at the Malawi Liverpool Wellcome (MLW) Programme, where only the study team had access. Electronic data (audio recordings and transcripts) were stored in duplicate in the MLW central data repository and Redcap in password-protected computers. After listening to each interview multiple times to comprehend the content, the interview was transcribed, translated, and read to confirm the audio. The transcripts were uploaded to Dedoose version 9.2.005 [24], a data management and analysis cloud application. The interview data were analysed using thematic analysis [25]. Initially, two authors (YC and CC) independently and deductively coded three transcripts using the three-delay framework and respectful maternity care domains. We employed a multiframework approach to identify delays and barriers to quality care for mothers with sepsis. The Three Delays model [26] is historically known as a well-established framework to examine delays in (1) seeking, (2) reaching, and (3) receiving appropriate and quality health care. The Respectful Maternity Care [27] framework’s domains were used to assess each patient’s journey to establish whether a health facility and its health providers meet women’s expectations of respectful and inclusive care [28]. The RMC domains are (1) no harm or mistreatment; (2) information, choice, and preference; (3) privacy and confidentiality; (4) dignity and respect; (5) equality and equity, no discrimination; (6) high-quality care; (7) liberty and autonomy, no arbitrary detention; (8) child with parents or guardians; (9) child identity and nationality from birth; and (10) adequate nutrition, clean water [14]. If a woman narrated suitably within any domain of these frameworks, we coded it accordingly. Inductive coding was used when narratives did not fit into either framework domain. After agreeing on the coding framework, meanings, and applications, the interrater reliability was confirmed for six transcripts, and the first author completed coding for the remaining transcripts. The second author randomly selected an additional two transcripts to code to maintain interrater reliability. Code patterns were used to establish themes during the analytic discussions. After choosing the themes, the participants were titled and explained to simplify the data. The themes with limited data were combined with similar or related themes while ensuring that the themes remained independent and that there was no duplication of information. We chose quotations representing the primary theme or subtheme to lend credibility and present the patients’ data in their own words. In discussions about data analysis, patient identification numbers were mentioned to refer to the women, avoiding any personally identifiable information.

### Ethical considerations

Ethical approval was obtained from the Kamuzu University of Health Sciences Research Ethics Committee (P.10/22/3793) and the University of Liverpool Central University Research Ethics Committee (UoL001719). Ethical approval for the parent study (The Lactate study) was obtained from the Kamuzu University of Health Sciences Research Ethics Committee (P.10/22/3553) and the University of Liverpool Central University Research Ethics Committee (UoL10737). All participants provided written informed consent and were informed of their right to terminate their participation at any time.

## Results

### Screening and enrollment

**Fig. 1:**
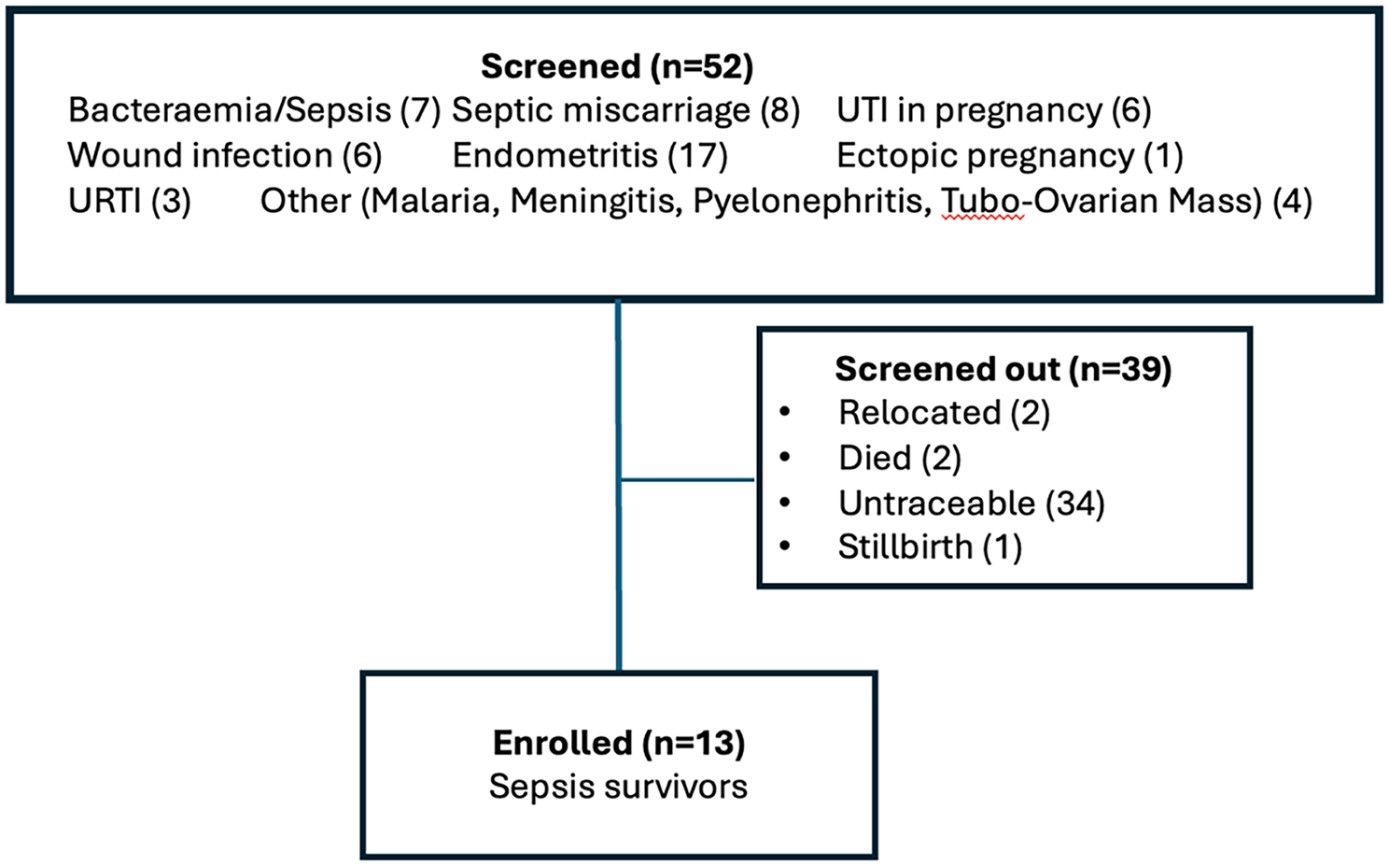
Screening and enrollment process for women who fulfilled the criteria for maternal sepsis

From a list of 52 eligible participants (Fig. 1), women were screened out if they had relocated outside of Blantyre, if they had died, if the contact information was insufficient to trace them by phone or physically, or if they had experienced a fetal or neonatal loss (as the recollection of loss could have negative mental health impacts on the mother), a total of 13 sepsis survivors enrolled in this study.

### Characteristics of the study participants

The age range was 16 to 37 years, and all the women lived in urban settings. Most were married women with secondary-level education who were housewives or businesswomen (Table 1).

**Table 1:** Characteristics of maternal sepsis survivors (N-13)

Characteristic	*N* (= 13)
**Age (years)**	16–37 years
**Marital status**	
Single Married Divorced	1 11 1
**Education level**	
Primary Secondary Tertiary	2 10 1
**Occupation**	
Student Housewife Domestic worker Business	1 7 1 4
**Current residence**	
Rural Urban	0 13

### Clinical presentation and outcomes of maternal patients

Most (11 out of 13) women were admitted in their postpartum period, while only 2 women were in the intrapartum period. The most common primary infection condition was endometritis, with 7 out of 8 maternal patients having had an emergency caesarean delivery due to obstructed labour or fetal distress. Severe maternal outcomes (any life-threatening condition requiring intensive care unit (ICU) or high dependency unit (HDU) admission, blood transfusion, laparotomy +/- hysterectomy, or evidence of one or more organ dysfunctions) occurred in 6 maternal patients in this study (Table 2).

**Table 2:** Clinical presentation and outcomes of maternal sepsis survivors

Characteristics	*N* (= 13)
**Pregnancy period**	
Antepartum Intrapartum Postpartum	0 2 11
**Primary infection suspected/confirmed.**	
Endometritis Pyelonephritis Peritonitis Septic miscarriage Deep wound infection	8 1 1 2 1
**Days hospitalized (days)**	10.5 (4–14) ^§^
**Severe maternal outcome (SMO)**	
Yes No	6 7

^§^ Range of days hospitalised

### Survivors of maternal sepsis

**Fig. 2:**
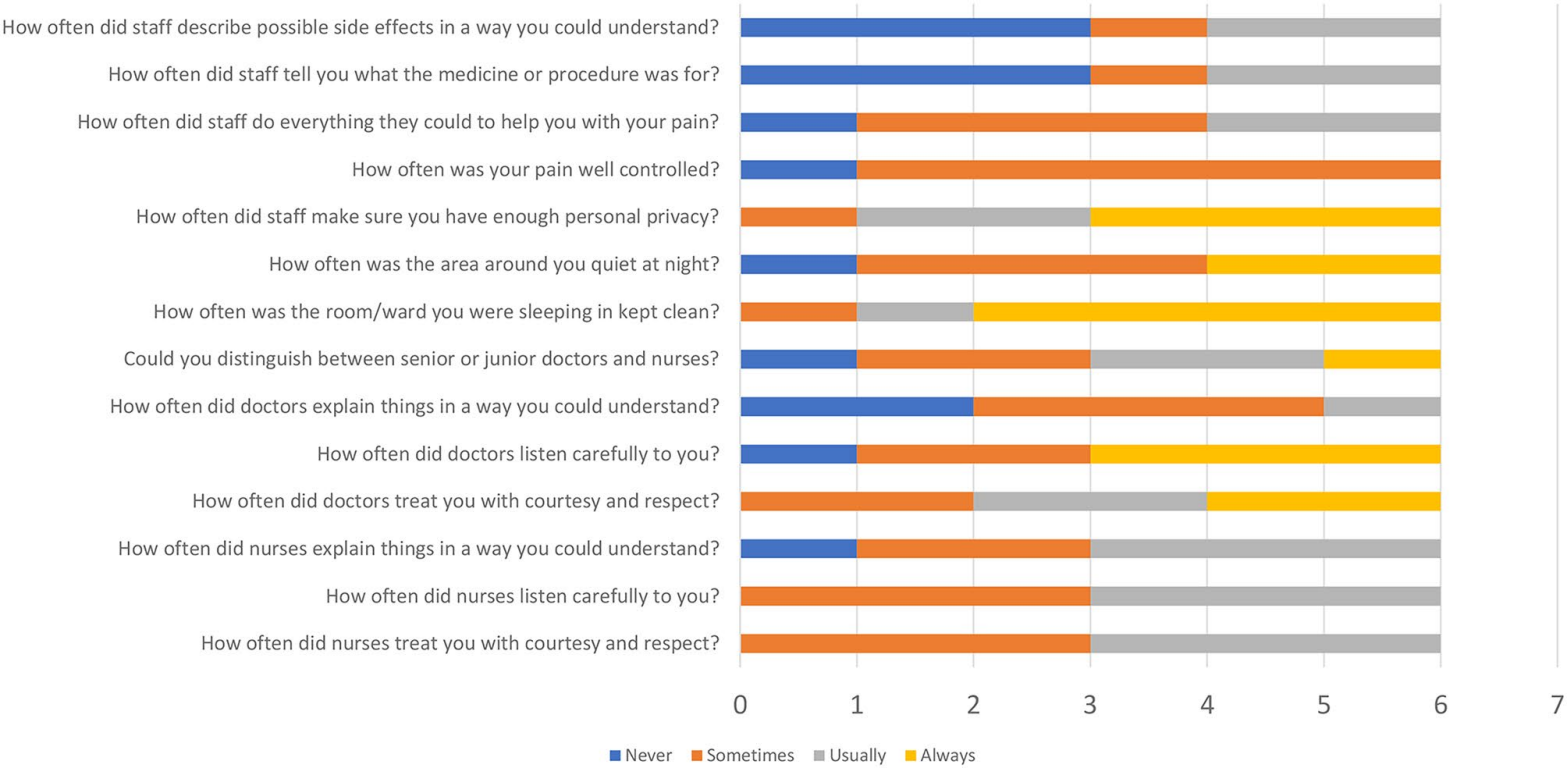
In-patient assessment of healthcare (I-PAHC)

**Fig. 3:**
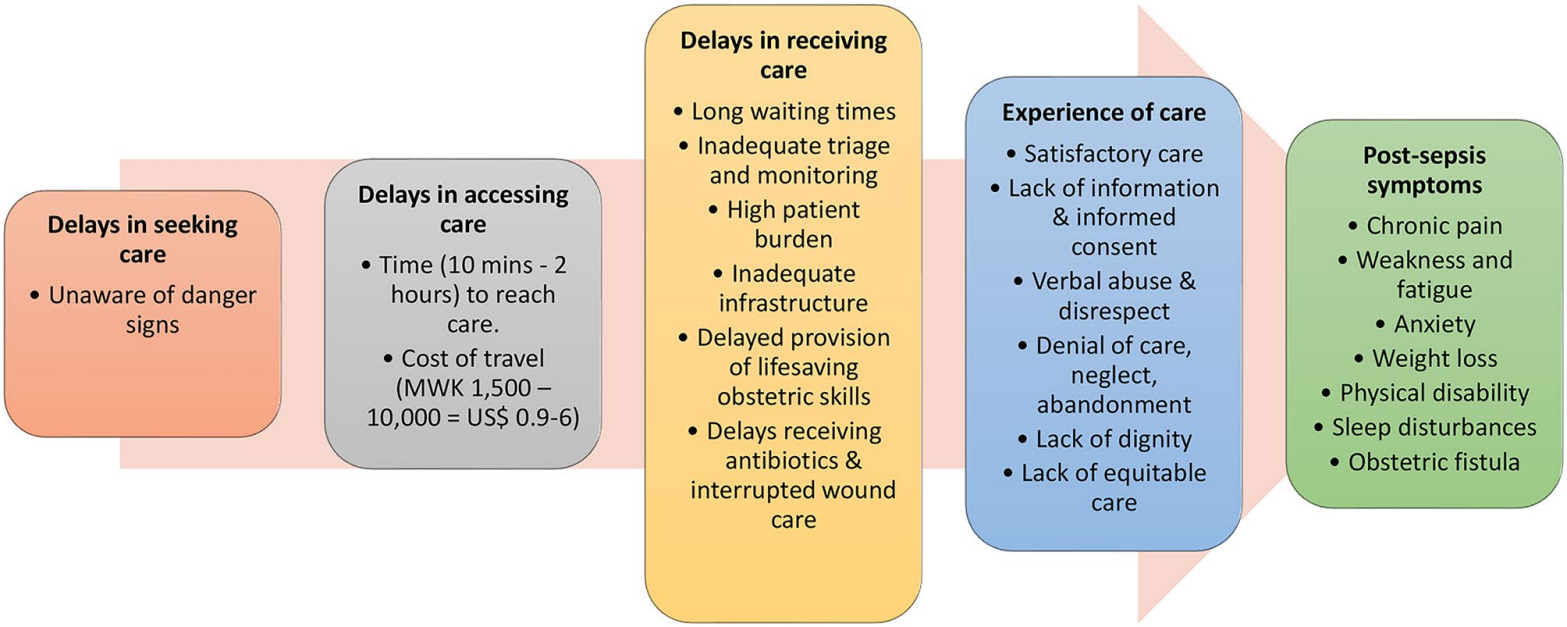
The five main themes make up a consolidated maternal sepsis patient journey. Within each theme are the reasons for delays, types of care experiences, and post-sepsis symptoms reported

Fig. 2 shows a selection of questions with responses from the I-PAHC questionnaire. During the qualitative analysis of in-depth interview data and responses from the I-PAHC questionnaire, findings related to delays in seeking, reaching, and receiving appropriate care (from the three-delay model), mistreatment, denial of care, neglect and abandonment, respect and dignity, and equitable care (from the respectful maternity care domain) were identified. Figure 3 summarises the delays in care, experiences of care, and post-sepsis sequelae in maternal sepsis survivors.

Fig. 2 presents a selection of questions from the IPAHC questionnaire, highlighting patients’ evaluation of their interactions with healthcare providers regarding treatment, procedures, and overall rapport

## Delays in seeking care

Among the women whose symptoms began at home, most delayed seeking medical attention for at least one day. Before presenting to a healthcare facility, some women attempted to rest or sought counsel from an elder (mother or grandmother), possibly indicating that they were unaware of the signs of infection.

*“It was Sunday*,* and I left to join my friends at church; I felt my body getting weak*,* and when I got home*,* I felt cold. I just went to the bedroom and fell asleep. That night*,* I was very restless and could not sleep. I started draining fluid*,* and when I got up to urinate*,* I saw something had fallen out [from my vagina]. Then*,* I left in the morning to go to the hospital”*. (23 years old, with a septic miscarriage)

*“I waited for two days [at home] to figure out what was happening but couldn’t see it. When I went to Machinjiri [health center] to explain on the first day*,* they gave me medicine to go back home; when I came home*,* I just drank the medicine and vomited. I took water and bathed*,* and I still didn’t feel well. That’s when I called my mother and grandmother*,* and they said it is important to return after I explained it to them.”* (25 years old, vaginal delivery, endometritis)

## Delays in reaching care

It took women 10 min to two hours to reach QECH from home or the health centre. Travel expenses to QECH ranged from 1500 to 10,000 Malawi Kwacha (MWK), translating to approximately US$ 0.9 to 6. This is notable considering an average daily income of US$ 2 for an urban worker. The women used a variety of modes of transportation, including ambulances, motorcycles, hired taxis, and public transportation. One woman who had been discharged after delivery was fortunate enough to have access to her sister’s car and returned to the hospital promptly once her condition worsened, requiring immediate attention. Her experience highlights that when timely access to healthcare is available, prompt recognition of the problem, triage, and immediate care is available at QECH.

“*The next morning*,* my mother came to check on me*,* and this time*,* when wiping the wound with cotton*,* the whole cotton ball got soaked with pus discharge coming from my incision. My mother quickly called my sister to come to the house. When my sister arrived*,* she commented that the entire bedroom smelled awful and pointed out many large black flies around me. They packed up*,* and my sister drove me back to QECH. On arrival*,* the nurse noted my condition and quickly taken me to the nurse’s office. I didn’t have to wait in line. The nurse opened up the incision and started cleaning and squeezing the pus while another nurse rushed to call a doctor to come attend to me*.” (32 years old, laparotomy for endometritis)

## Delays in receiving appropriate care

When women arrived at QECH, almost all encountered delays in receiving proper care; delays varied from the timing of simple triage and vital sign checks to when medication was given (contrary to the prescribed frequency) to how long women had to wait to receive definitive operative interventions (dilation and curettage or emergency cesarean section). Women reported long wait times in the hospital admission room and, while in the ward, waiting from 4 to 24 hours before receiving appropriate care. Some women stated that no vital signs or fetal monitoring was performed while they waited for assistance. Women in labor had to wait for beds in the labor ward to become available before receiving assistance. Women who had been referred to QECH due to labor complications had long wait times despite being a high priority on arrival. Some women complained about the lack of order at the admissions desk, feeling like they were being ignored or passed over despite being next in line for treatment.

“*I waited for some time at the reception. You’d find they were helping those who found me there waiting*,* assisting them first instead of me. Then*,* I spent 12 hours in the labor ward*,* waiting. My waters broke at approximately 1 am in the early hours. From that time*,* I waited until I went to the theatre at 5 pm. When my waters broke*,* my sister went to tell the nurse to come and check on me and the baby. I could see that there was a baby’s feces in the fluid. When the nurse checked me*,* she also told me that the baby had made feces inside my uterus. When her cervix was checked*,* she found that the cervix had closed again and was 3 cm long. The nurse said she needed to discuss it with her fellow doctors*,* and then she would come back and tell me the way forward. In addition*,* then*,* after some time*,* she came to tell me that I would need to go for an operation. They came to put those drips on me*.” (24 years old, emergency C-section due to fetal distress)

“*I arrived in the labour ward at the past 3 pm*,* and they told me I would need to wait until 7 pm*,* and if I had not delivered by then*,* then I would go to the theatre. By the time it was 7 pm and they were coming to get me to go to the theatre*,* they told me to wait first because another woman was bleeding heavily and needed to go into the theatre now*,* and I should wait. Therefore*,* I waited until the nurse returned after some time to say I needed to wait again for another woman to go into the theatre before I could go. While I was still in the ward waiting to go to the theatre*,* and with how I felt*,* I called out to a nurse passing by. She checked me and said the way things were looking*,* the baby’s head was near the exit*,* so I had to lie on my side and keep pushing*,* and then she left us alone. At that point*,* I can say the care was bad. When I went to the theatre*,* it was past 1 am*,* and the baby was born approximately 4 am” (18 years old*,* emergency C-section due to obstructed labour*,* peritonitis and severe sepsis*,* ICU admission)*

## Experience of care

### Subtheme one: satisfactory care in an average health facility

Most (6 out of 13) women indicated their care was satisfactory and felt relieved when discharged. Most women had never been hospitalised at QECH before, and most of those who completed the I-PAHC questionnaire (6 out of 13) rated the facility below average. Most women indicated that they would refer QECH to a friend for care. However, one woman said she would never recommend QECH to a friend for care.

“*I just thanked the heavens that they were taking good care of me because some of the other patients complained that they were taking more care of me*,* and yet the rest of them did not have doctors come to take care of them as they did me. Therefore*,* I am happy that I was truly being taken care of*.” *(22 years old*,* septic miscarriage)*

### Subtheme two: women did not receive information or an explanation about their condition or treatment

Some women were not informed of their diagnosis or given a clear explanation of the cause of their illness. Some women said they never asked questions, while those who did were often not given important information, such as why they needed to stay in the hospital or what signs to watch for after discharge. Many women were given medicines that they had never taken before, yet half of them did not know what the medicines were for or what potential side effects they had. One woman was left feeling suspicious and had unanswered questions, believing that information about the cause of her severe infection was being concealed.

“*I didn’t know the reason why they were keeping me [in the hospital]. I was wondering myself because other patients would come and then get discharged*,* leaving me still in the hospital. Even trying to read your file was tedious. I kept telling myself I’d also get discharged soon.*” *(24 years old*,* emergency c-section for fetal distress*,* endometritis)*

*“My thoughts then were that maybe a mistake had happened at the hospital. I still had hope and faith. I told myself something was wrong*,* but I’ll be fine. Even though they never said it*,* the people at the hospital knew something was wrong when discharging me. I believe the problem began at the hospital while I was there*,* specifically during the caesarean section. I was afraid. I did not understand the journey of my wound becoming so infected as it did. As a patient*,* I believe patients and doctors are supposed to be on the same page. However*,* I still have questions about how the infection got so bad*,* and within such a short time*,* barely seven days*,* it was exactly seven days from my c-sections for me to be in such a bad situation.” (32 years old*,* laparotomy for endometritis)*

### Subtheme three: women who experienced verbal abuse by healthcare providers

Most (10 out of 13) women reported having satisfactory interactions with healthcare providers. However, one woman recalled an interaction with a nurse that qualifies as verbal abuse. She was chastised and mocked when she requested additional pain medicine. Because this woman did not hold back from her questions, she was verbally threatened with withholding treatment.

*“There were some [hospital staff] I got along with well who were understanding and helpful when I was in pain or had issues. Others were rude*,* just rude. There was one day when I wasn’t feeling well*,* so I asked for some medication. After more than an hour*,* a different nurse approached me to say*,* “In the hospital*,* we don’t beg for medicine; it’s like you are undermining the professionals taking care of you.” When I asked if I was wrong to say something or ask for help*,* I was told*,* “You see*,* that’s exactly why you won’t be helped because it’s like you are argumentative and needy” (32 years old*,* laparotomy for endometritis)*

### Subtheme four: women were denied care, neglected and abandoned

All the women reported experiencing pain during their hospital stay. The pain was usually well controlled, and hospital staff often provided medication. However, one woman was repeatedly ignored and denied care by unsympathetic nurses who were asleep at their duty station during the night shift. Most women felt that their grievances and concerns were acknowledged. However, some women complained that healthcare staff were dismissive of their complaints of unwellness, even when labeling them pretentious. Others reported being abandoned to the point of deterioration from an initially stable condition.

“*It was approximately 11 pm*,* and they were not coming to assist me. I overheard them in my neighbor’s room; she was being prepared for theatre when I called out to them for help*,* and during all this*,* I was in the room. I called for help*,* but they showed no interest in helping me. I was worried that they were not feeling sorry for me when I was in pain. I followed them into their working station with a drip in my arm*,* but they never attended to me. They were sleeping at the nurse’s station*,* saying they were not assigned to my room to help me. I should still wait*.” *(25 years old*,* emergency c-section for obstructed labor*,* endometritis)*

“*Some doctors were talking to me nicely when they saw I had an issue. Others were saying I was pretending and to leave me* alone” *(16 years old*,* pyelonephritis)*

“*I was upset because I was in terrible pain. By the time they were helping me*,* I was feeling even worse. When I arrived*,* I was disappointed with the length of time it took to be helped. When the healthcare worker finally came to assess me*,* I was not well at all. I was not satisfied with the care*.” *(23 years old*,* emergency C-section for obstructed labour*,* endometritis)*

*There was another day when my wound was oozing a lot*,* and the dressing even came off. I went to a nurse to tell them and asked if they could redress the wound. I was not helped. Some nurses responded rudely to me*,* but one nurse kindly said she would come to my bedside to redress the wound. Some hours went by*,* and when I reminded the nurse*,* she said*,* ‘Sorry*,* I forgot*,* I’ll bring them soon*,*’ but she didn’t come*,* and that night*,* I slept with an undressed open wound until morning.” (32 years old*,* laparotomy for endometritis)*

### Subtheme five: women who experienced disrespectful and undignified care

During their hospital stay, most women (9 out of 13) said that healthcare providers treated them with politeness and respect at times, while others were treated courteously and respected almost regularly. Most women believed that doctors treated them with decency and respect. However, one woman felt humiliated by the insulting remarks she received after a healthcare provider incorrectly labelled her as unclean when her infected wound was producing an offensive odour.

*“It’s just that the dressings they put on me got soiled*,* causing me pain. When I went to tell the nurse that the dressings were wet*,* they responded*,* “Oh no*,* you’re smelling*,* go and bath.” When*,* in fact*,* the problem was the pus from the wound and not because I hadn’t bathed. Therefore*,* no one will receive that kind of talk well. Because I knew the problem came from the wound and the soiled dressings.” (32 years old*,* laparotomy for endometritis)*

### Subtheme six: women lacked equitable care

Medications such as analgesics were freely available to all women while they were hospitalised. However, women (3 out of 3) who needed regular wound cleansing had to eventually purchase extra antiseptic cleaning solutions once the hospital supply ran out. For one woman, her incision was cleaned for a few days with hospital-provided supplies before she had to buy more to keep it clean. She was also prescribed an antibiotic that was expensive and difficult to find, causing a barrier to access and continuity of essential medications. This potentially jeopardised her care and instilled worry in the patient.

*For example*,* when they opened my incision to clean my wound*,* they said there was no medicine to continue cleaning*,* so they wrote me a prescription to go and buy the medicine. My sister went to buy it. They even wrote about an antibiotic for me*,* which was unavailable in the pharmacy. I was told that the antibiotic was scarce*,* that not enough was ordered and that it was expensive. Some days*,* I’d go the whole day without this antibiotic. I was afraid because we are always told that antibiotics need to be taken every day until the whole dose is completed. Therefore*,* being in my situation*,* I was very concerned about what would happen*,* what would happen to me next” (32 years old*,* laparotomy for endometritis).*

## Post-sepsis symptoms

We identified several long-term impacts of sepsis, including chronic pain, weakness and fatigue, weight loss, anxiety, physical disabilities, and sleep disturbances, all of which impair some women’s capacity to work and perform daily tasks. An unresolved obstetric fistula significantly impacts one woman’s sex life.

“*I can’t take care of myself like I used to before. Much of the work I’m meant to be doing has stopped*.” (23 years old, emergency c-section for obstructed labour, endometritis)

*“I sometimes wake up feeling weak and unable to work*” (26 years old, septic miscarriage)

“*I’m only doing light tasks right now. I haven’t started bending over*,* I should say*,* not bending over all the way. I haven’t bent over yet”* (24-year-old, emergency c-section for fetal distress, endometritis)

“*What has changed is [breaks down crying]; during delivery*,* they cut my bladder*,* but they sowed it back together. Therefore*,* when I’m having sex with my husband [pauses crying and catching her breath] …during sex*,* when you are supposed to have vaginal discharge coming out*,* urine starts coming out. In addition*,* this still happens until now*.” (18 years old, emergency c-section due to obstructed labour, peritonitis and severe sepsis, ICU admission)

## Discussion

This study aimed to explore the experiences of maternal sepsis survivors in Malawi to influence sepsis care and management. We found that women most frequently experienced a delay in receiving adequate and appropriate care at the health facility level. Although most women had a positive experience of care, some women’s experience of maternal sepsis was compounded by exposure to mistreatment, mainly verbal abuse, neglect and abandonment, disrespect, denial of care and inequitable care. Some women continue to experience long-term physical and psychological complications from maternal sepsis.

Our finding that the third delay was the most frequent delay experienced by maternal sepsis survivors is consistent with the findings of several studies that also applied the Three Delays framework to other maternal health issues. Mgawadere et al. reported that 94.7% of maternal deaths at Mangochi District Hospital in the southern region of Malawi involved delayed treatment upon admission; healthcare providers attended to only 50.6% of women within 30–60 min of arrival at the facility, and 56.5% of women’s care lacked necessary antibiotics [29]. In Mozambique, 69.7% of maternal deaths are linked to the third delay [30]. In Ethiopia, the third delay is experienced by 74.7% of pregnant women with severe maternal outcomes [31]. The third delay typically indicates substandard quality of care at a health facility and weaknesses in the healthcare system. Care provision at this level relies heavily on healthcare providers’ attitudes and competencies, availability of medical supplies, and adequate management [26]. Addressing third delays requires a multi-faceted approach focusing on various areas of the health system. For example, a priority is to strengthen facility conditions by ensuring adequate staffing, resources, and infrastructure. Secondly, fully implementing and adhering to national quality standards and protocols to keep the health system operational. Thirdly, building healthcare provider capacity by increasing the health workforce and providing continuous training in respectful and evidence-based care [32]. Given the significant fiscal constraints facing the health sector in Malawi [20], it is essential to concentrate on cost-effective and evidence-based intervention strategies to mitigate the third delay, enhance infection management, and alleviate the disease burden associated with maternal sepsis in Malawi. We can learn from the Saving Mother, Giving Life (SMGL) initiative in Zambia and Uganda [32]. It successfully implemented systemwide strategies to reduce deaths due to the third delay by providing timely and appropriate delivery care for women and their newborns. Within five years, the initiative renovated and upgraded operating theatres, increased delivery beds, and refurbished rooms to enable an increased volume of facility deliveries and more extended postpartum stays; procured essential medications; implemented logistics management systems to reduce or eliminate stock-outs; recruited sufficient numbers of healthcare providers to meet national standards for comprehensive emergency obstetric and neonatal care (CEmONC) facilities; and provided tools, guidelines, and policies to ensure protocol adherence, all aimed at improving the quality of care. These key strategies significantly reduced maternal mortality in Uganda and Zambia [32]. Malawi could adopt these findings and implement similar interventions and approaches to mitigate avoidable sepsis-related severe maternal outcomes and to avoid a decline in the significant improvements made in access to healthcare services.

In our study, the women’s experiences reflected that the care provided did not meet the standard of respectful maternity care. Women were poorly informed about their condition and treatments; they experienced verbal abuse, denial of care, neglect, and disrespect by healthcare providers in the maternity wards. Multiple studies have demonstrated that women across the world have experienced mistreatment during childbirth at health facilities [33–39]. Furthermore, evidence shows that women’s experiences of care during labour, birth and early postpartum influence their intention to seek maternity care in the future [40, 41]. To improve the outcomes of maternal sepsis during pregnancy or postpartum and ensure that women continue to seek maternity care for future pregnancies, the provision of care needs to maintain dignity, privacy, and confidentiality; ensure freedom from harm and mistreatment; and enable informed choice and continuous support for maternal patients during and after sepsis [40, 42]. Simply reinforcing the informed consent process for women undergoing all medical interventions at health facilities would allow patients to safeguard themselves from unwelcome procedures, maintain their autonomy, and uphold their moral and legal rights [43]. Implementing routine training or a continuous development program for healthcare providers focused on respectful maternity care, effective communication and patient-centered care is crucial. Embedding refresher courses as mandatory periodic certifications would ensure an ongoing commitment to staff development. This approach ensures maternal patients feel heard and respected during their care. We’d also recommend training healthcare providers and managers to proactively identify patient barriers, improve care pathways, and foster a culture of continuous quality improvement. Sufficient mentoring and supportive on-the-job training have been shown to help healthcare providers deliver effective reproductive, maternal, and newborn care [44]. At the community and public levels, building public demand for high-quality maternity services that provide women-centered care, educating women about their health needs and rights, and empowering them not to tolerate mistreatment have been effective strategies in other settings [45]. Despite the multitude of health system constraints that negatively impact the quality of care provided in low-resource settings, such as Malawi, women’s experience and perception of care need to be validated and considered vital indicators of the quality of the health system’s care. Health systems must be accountable, and sufficient resources must be available to provide quality, accessible maternal healthcare with clear policies on women’s and patients’ rights [45]. Our approach to investigating maternal sepsis patient journeys was intentional, as there was a clear gap in well-documented patient experiences of care in Malawi’s maternal service delivery. We aimed to foster learning and encourage open discussions about patient care using these patient journeys, which can potentially improve maternal healthcare. Concentrating solely on the patient’s experiences without considering input from healthcare professionals remains valuable for understanding the challenges in maternal healthcare. Yet, it does not provide a complete picture of the quality of care. Otherwise, the perspectives presented in this study would be biased and incomplete. We propose that quality improvement solutions incorporate patient experiences, clinical perspectives, systemic challenges, and resource constraints. This does not imply that women’s perspectives are invalid; instead, they should be complemented with a broader consideration of healthcare workers’ views, facility-level settings, and, more generally, the local health system.

Our research appears to be the first to document post-sepsis syndrome in Malawi, highlighting some long-term physical and psychological symptoms following sepsis [46]. In Malawi, there exists a significant knowledge gap regarding this condition, primarily due to insufficient post-discharge follow-up to detect and identify these symptoms and the recovery or rehabilitative needs of women who have faced severe consequences from sepsis. We advocate for more proactive investigations of post-sepsis symptoms across all patient demographics to define care needs that can shape healthcare policy, particularly in low-resource settings.

We focused on sepsis-related experiences. However, many lessons learned from these patient journeys may be relevant to care for all critically ill women, and the methodology we employed could be used and applied more widely in women’s health. Our study highlights the continued value of applying the three-delay model to identify barriers to women receiving necessary maternal health care. We recognised early in the design stage of this study that the delay framework would not sufficiently capture all aspects of barriers to maternal care; therefore, using the concepts of respectful maternity care, an area with limited data from Malawi, we could further describe the experiences and quality of care in maternal healthcare.

### Study limitations

This study has several limitations. Our research on patient journeys is based on a single tertiary hospital, where it just so happens that most of the women interviewed were married housewives with secondary-level education who lived in urban environments. Therefore, these care experiences cannot be generalized to all women who have encountered negative journeys in care. We used a rigorous and academically robust process to obtain high-quality data, which required specialist expertise and formal approval. This makes such a data collection process for patient experiences challenging to scale across different facility settings and diseases. We purposively recruited women to participate in this study, aiming to gather diverse maternal sepsis experiences with differing pregnancy outcomes at different stages of pregnancy. Nevertheless, with the goal of diversity in stories, we may have underestimated delays or mistreatment due to selection bias. A formal yet simplified process is necessary to gather patient perspectives to contribute to health policy and health service reform. The patient journey mapping approach is increasingly being implemented in developed nations to enhance the responsiveness and transparency of health systems [47]. Context-appropriate feedback mechanisms must be explored to ensure regular input of patient perspectives and experiences into hospital quality systems in Malawi.

### Policy implications

Implementing a policy mandate for health facilities to collect and analyse patient narratives, especially from women who have faced sepsis experiences [48], may help identify care gaps and guide specific improvements in infection and sepsis management. Formal methods like exit interviews or focus groups could be used to gather patient feedback regarding sepsis care, addressing issues such as delays, miscommunication, or barriers to receiving treatment. Additionally, incorporating satisfaction metrics and qualitative insights into the monitoring frameworks of maternal health programs would enable regular evaluations of how policy changes affect patient care experiences [49].

### Implications for future research

Future studies examining patient journeys in district and primary-level facilities could offer additional insights into the experiences of care for the entire healthcare system. Additionally, investigating the phenomenology and epidemiology of post-sepsis syndrome in low-resource settings would generate evidence and better inform home or community-based healthcare needs.

## Conclusions

Women’s experience of maternal sepsis often involves delays and insufficient care in healthcare settings. While many can recount positive experiences, others have faced different forms of mistreatment, such as verbal abuse, neglect, and inequitable treatment. Furthermore, some women continue to endure lasting physical and psychological effects of maternal sepsis. Understanding women’s experiences and perceptions of care is crucial for measuring quality and gathering evidence to improve sepsis prevention and management in health facilities. Patient narratives of near-miss experiences can inform policy and practice to improve maternal healthcare. This study’s findings are highly applicable to other lower-middle-income countries facing high rates of maternal infections and sepsis within the context of resource-constrained healthcare systems. To meet global maternal health objectives, comprehensive patient-centered interventions that enhance the management of maternal infections resulting in sepsis are essential. These interventions will help reduce preventable maternal morbidity and mortality.

## Supplementary Information

Below is the link to the electronic supplementary material.

Supplementary Material 1

Supplementary Material 2

## Data Availability

No datasets were generated or analysed during the current study.
