# Supplementary material for: Understanding the maternal sepsis patient journey in Malawi: “I called for help, but they showed no interest in helping me”
Source: BMC Health Serv Res. 2025 Sep 30;25:1246. doi: 10.1186/s12913-025-13459-1 (PMC12482413; doi:10.1186/s12913-025-13459-1)
Supplement: Supplementary file 1 — Supplementary Material 1 [file 12913_2025_13459_MOESM1_ESM.docx]

Appendix 1: In-patient Assessment of Healthcare (I-PAHC) – Participant responses

| **Q** |  | **Never** | **Sometimes** | **Usually** | **Always** |
| --- | --- | --- | --- | --- | --- |
| 1 | During this hospital stay, how often did nurses treat you with courtesy and respect? |  | 3/6 | 3/6 |  |
| 2 | During this hospital stay, how often did nurses listen carefully to you? |  | 3/6 | 3/6 |  |
| 3 | During this hospital stay, how often did nurses explain things in a way you could understand? | 1/6 | 2/6 | 3/6 |  |
| 4 | During this hospital stay, how often doctors treat you with courtesy and respect? |  | 2/6 | 2/6 | 2/6 |
| 5 | During this hospital stay, how often doctors listen carefully to you? | 1/6 | 2/6 |  | 3/6 |
| 6 | During this hospital stay, how often doctors explain things in a way you could understand? | 2/6 | 3/6 | 1/6 |  |
| 7 | Could you distinguish between senior or junior doctors and nurses? | 1/6 | 2/6 | 2/6 | 1/6 |
| 8 | During this hospital stay, how often was the room/ward you were sleeping in kept clean? |  | 1/6 | 1/6 | 4/6 |
| 9 | During this hospital stay, how often was the area around you quiet at night? | 1/6 | 3/6 |  | 2/6 |
| 10 | During this hospital stay, how often did staff make sure you have enough personal privacy? |  | 1/6 | 2/6 | 3/6 |
| 11 | During this hospital stay, did you experience any pain? | Yes  6/6 | No |  | |
| 12 | During this hospital stay, how often was your pain well controlled? | 1/6 | 5/6 |  |  |
| 13 | During this hospital stay, how often did staff do everything they could to help you with your pain? | 1/6 | 3/6 | 2/6 |  |
| 14 | During this hospital stay, were you given any new medication (medication you had never taken before)? | Yes  5/6 | No  1/6 |  | |
| 15 | Before giving you any new medication, how often did staff tell you what the medicine or procedure was for? | 3/6 | 1/6 | 2/6 |  |
| 16 | Before giving you any new medication, how often did staff describe possible side effects in a way you could understand? | 3/6 | 1/6 | 2/6 |  |
| 17 | Were you given information in a way you could understand what symptoms or health problems to look out for after you leave the hospital? | Yes  4/6 | No  2/6 |  |  |
| 18 | Was it easy to find your way around the hospital? | Yes  3/6 | No  3/6 |  |  |
| 19 | Is this your first time being treated at this hospital? | Yes  5/6 | No  1/6 | Cannot remember | |
| 20 | On a scale of 0 – 10 (0 being the worst hospital, 10 being the best hospital), how would you rate this hospital? | 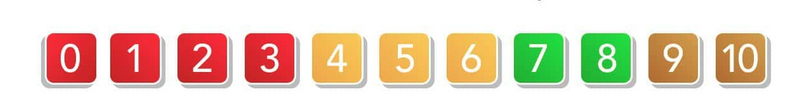  3/10 (1); 4/10 (3); 5/10 (1); 7/10 (1) | | | |
| 21 | Would you recommend this hospital to your friends and family? | Definitely no  1/6 | Probably no | Probably yes  3/6 | Definitely yes  2/6 |
| 22 | Did you have to pay for anything (drugs, supplies, tests, treatment, pay staff, food) during this hospital stay? | Yes  1/6 | No  5/6 | If yes, specify: drugs | |
| 23 | Did you have to pay for anything outside the hospital during your stay? (e.g., costs at home (rent, bills), childcare, food to be brought to you, etc) | Yes | No  6/6 | If yes, specify. | |
| 24 | How do you consider this hospital and healthcare experience? | Poor | Fair  5/6 | Good | Excellent  1/6 |
