# Supplementary material for: Understanding the maternal sepsis patient journey in Malawi: “I called for help, but they showed no interest in helping me”
Source: BMC Health Serv Res. 2025 Sep 30;25:1246. doi: 10.1186/s12913-025-13459-1 (PMC12482413; doi:10.1186/s12913-025-13459-1)
Supplement: Supplementary file 2 — Supplementary Material 2 [file 12913_2025_13459_MOESM2_ESM.docx]

Appendix 2: In-depth Interview Guide – English

Interviewer instructions:

- The interviewer introduces self and thanks the participant(s) for sparing time for the interview.
- Introduce the study – brief background and objectives of the study. Describe clearly the research and the role of the participants, the commitment involved, any foreseeable risks, and expected benefits.
- Get written consent—Ask whether the participant has understood the study and is willing to sign the consent form. If they are willing to sign it, proceed to the next step.
- Getting to know the interviewee: Ask how she feels today before the 1.5–2 hour interview. If she feels well enough and can spare the time, proceed to the next step.
- Allow the interviewee(s) to speak as much as possible about the event during the interview.
- Return to the story's beginning and ask follow-up and probing questions to clarify.
- At the end of the interview, thank the participant for their full participation and time.

| **Interview topics** | **Questions** | **Probes** |
| --- | --- | --- |
| About you | 1. How would you describe your health before this hospital admission? | - Overall health, work, and family situation. - Any personal characteristics that might impact your health (previous bad obstetric history, chronic   conditions, age, poverty) |
| Prevention | 2. What sorts of health services do you use? | - Local dispensary - Community Health worker/Health surveillance assistant - Advice from an elder - Traditional healer - Self-care - home remedies - Health centre/hospital staff |
| Recognition of danger signs | 1. When did you start feeling unwell? 2. What did you (or others) feel was wrong with or was the cause of the problem? 3. How did you feel? | - Progression of symptoms and signs (what did you feel at first, what was the last feeling before seeking help?) - Date of onset of symptoms (estimate) - Onset of symptoms (gradual or sudden - Feelings of optimism (I will be ok) - Feelings of fear (This is serious, I will not be ok) - Feelings of anxiety (I don’t know what   will happen to me)   - Feeling no concern (This is nothing to worry about) |

| Seeking health assistance | 1. When you noticed something was wrong with your health, what did you do about that, if anything? 2. Where did you go for help? | - Seek advice from spouse/partner, family/relatives, community members, health professional (HSA, health centre staff, hospital) - Decision makers - Delays in decision making |
| --- | --- | --- |
| Access to care | 8. How did you get to the hospital? | - First point of contact with health system. - Stops along the way or referral points. - Distance and time to get to hospital - Geographical issues (terrain, weather) - Costs and mode of transport - Social & family costs to leave home. - Guardianship - Emotions and feelings during travel to hospital or during referral points. |
| Diagnosis | 1. What happened on arrival at the facility? 2. Can you tell me about how you came to be diagnosed? | - Waiting times before being assisted a health professional - Diagnostic tests and imaging - Being told the diagnosis - Hospitalisation and/or treatment as an outpatient |
| Treatment | 11. Tell me about your treatment. | - Medications/treatment given - Interaction with nurses and doctors – attitudes, behaviour, empathy from staff - Quality of care – satisfied or not. - Lack of supplies (medications, diagnostic tests, health personnel) - Costs of care - Autonomy during care - Mental well-being during care. |
| Discharge | 12. How did you feel when you were told you were discharged from hospital? | - Feelings (joy, excited, relief, fear, anxiety, regrets) - Impacts on work/community/family and home life. |
| Continuity of care | 1. What happened after you left the hospital? 2. Who looked after your health? 3. Did the hospital or other services follow up once you were back home? 4. Are you continuing and medications or self-care since being discharged? | - Journey back home - Family support - Follow-up plan |
| Post-sepsis | 1. Has much changed in your life because of your condition? 2. Do you do anything different now, to look after your health? 3. If a friend of yours was diagnosed with the same condition, what would you tell them to prepare for? 4. If you could change one thing about your care, what would that be? | - Life after sepsis (family, social, sexual life) - Overall impressions - Key recommendations for change - What mattered most to you about this entire experience with sepsis? |
